# Reevaluation of the evolutionary events within recA/RAD51 phylogeny

**DOI:** 10.1186/1471-2164-14-240

**Published:** 2013-04-10

**Authors:** Sree V Chintapalli, Gaurav Bhardwaj, Jagadish Babu, Loukia Hadjiyianni, Yoojin Hong, George K Todd, Casey A Boosalis, Zhenhai Zhang, Xiaofan Zhou, Hong Ma, Andriy Anishkin, Damian B van Rossum, Randen L Patterson

**Affiliations:** 1 Department of Physiology and Membrane Biology, School of Medicine, University of California, Davis, USA; 2 Department of Biochemistry and Molecular Medicine, School of Medicine, University of California, Davis, USA; 3 Center for Translational Bioscience and Computing, University of California, Davis, USA; 4 Center for Computational Proteomics, The Pennsylvania State University, Pennsylvania, USA; 5 Department of Biology, The Pennsylvania State University, Pennsylvania, USA; 6 Department of Computer Science and Engineering, The Pennsylvania State University, Pennsylvania, USA; 7 Molecular, Cellular and Integrative Physiology Graduate Group, University of California, Davis, USA; 8 Department of Biochemistry and Molecular Biology, The Pennsylvania State University, Pennsylvania, USA

**Keywords:** Recombinase, recA, RAD51, Phylogenetic inference

## Abstract

**Background:**

The recA/RAD51 gene family encodes a diverse set of recombinase proteins that affect homologous recombination, DNA-repair, and genome stability. The recA gene family is expressed across all three domains of life - Eubacteria, Archaea, and Eukaryotes - and even in some viruses. To date, efforts to resolve the deep evolutionary origins of this ancient protein family have been hindered by the high sequence divergence between paralogous groups (i.e. ~30% average pairwise identity).

**Results:**

Through large taxon sampling and the use of a phylogenetic algorithm designed for inferring evolutionary events in highly divergent paralogs, we obtained a robust, parsimonious and more refined phylogenetic history of the recA/RAD51 superfamily.

**Conclusions:**

In summary, our model for the evolution of recA/RAD51 family provides a better understanding of the ancient origin of recA proteins and the multiple events that lead to the diversification of recA homologs in eukaryotes, including the discovery of additional RAD51 sub-families.

## Background

recA/RAD51 is an ancient protein family that evolved to perform diverse roles in DNA management. These roles include repair, recombination, and maintenance of genome stability [1-3]. There are three accepted subfamilies: recA, RAD*α*, and RADβ [4-8], and these can be further subdivided into additional clades that have specific functions. For example, bacterial recA is a DNA-dependent ATPase that binds to single stranded DNA to promote homologous recombination; in eukaryotes, these functions are performed by RAD51 members [9-11]. Knock-out of recA in bacteria leads to cell death due to the accumulation of deleterious mutations [12]. Similarly, RAD51 knock-out mice exhibit cell death and embryo inviability [13]. DMC1, a eukaryote specific group, is required for meiotic recombination [14] with DMC1 knock-out mice manifesting truncated oogenesis. Therefore, taken as a group, recA/RAD51 proteins are of fundamental importance for cell-viability across all domains of life. More importantly, duplications of ancestral recA sequences and diversification of functions led to the increased complexity apparent in extant species [7,15].

Seminal phylogenetic studies on this superfamily by Lin *et al.*[16] proposed that: (i) bacteria contain only one recA gene, (ii) archaea contain two recA genes (RADA and RADB), (iii) yeast have four recA genes, and (iv) vertebrate animals and plants have at least seven recA genes [4,5,10,11]. These studies provided considerable support for orthologous groupings for recA, RADA, RADB, DMC1, RAD51, XRCC2, XRCC3, and RAD51B-D (see Additional file 1 Figure S1A for representation of their phylogenetic inferences), and led to the postulate that eukaryotic recA genes evolved via two independent endosymbiotic transfer events. However, to obtain these groupings, several highly divergent sequences were omitted from the analysis because of their ambiguous placement in the tree.

More recently, Wu *et al.*[17] used a metagenomic survey approach to isolate a number of potentially ancient members of the recA family (i.e. recA-SAR1, Phage UvsX, Phage SAR1, Phage SAR2, Unknown 1, and Unknown 2). From this analysis, they concluded that: (i) these sequences are related to the recA/RAD51 protein family, (ii) several of these new groups are either viral lineages (e.g. bacteriophage) or archaeal in origin, and (iii) one new group, designated Unknown 1, is very distant from the other groups and may belong to a fourth domain of life. Wu *et al*. [17] also identified Unknown 1 as an metagenomic sequence with no useful information with respect to its sequence origin, which branches deeply (i.e. either between the three domains or as one of the deepest branches within a domain). Although these findings are potentially of great importance, the phylogenetic trees including these metagenomic sequences differ from those of Lin *et al.*[16]. In particular, the branching pattern of archaeal sequences, occupying a key place in the history of recA recombinases, differs between these studies (compare Additional file 1 Figure S1A and S1B).

To discriminate between these two disparate phylogenetic results, we applied our recently developed Position Specific Scoring Matrix (PSSM)-driven algorithm, termed PHYlogenetic ReconstructioN (PHYRN), that is highly accurate and robust for tree inference in highly divergent protein families [18]. PHYRN was benchmarked in simulated data sets with average pairwise identity <8.5% and was shown to be more accurate than multiple sequence alignment using either Maximum Likelihood [19] or Bayesian [20] methods. PHYRN can handle large and diverse data sets, which may be required to discriminate between phylogenies proposed by Lin *et al.*[16] and Wu *et al*. [17]. This study describes PHYRN-based estimates of deep phylogenetic relationships within the recA/RAD51 superfamily and compares the tree branching pattern, statistical support, and evolutionary inference by PHYRN pipeline to the data sets representative of the Lin *et al.*[16] and Wu *et al.*[17] studies. From the combined data, we propose a model of recA/RAD51 evolution that: (i) includes more diverse members of recA/RAD51 lineages and the new basal groups isolated by Wu *et al.*[17] from metagenomic sources, (ii) largely accords with the overall general pattern of Lin *et al.*[16], (iii) identifies new RAD51 paralogs that share commonalities between RADA and RADB, and (iv) lends support to the idea of the basal origin and diverse nature of metagenomic sequences as proposed by Wu *et al.*[17]. Taken together, our findings further resolve the deep origins of recA/RAD51 family and demonstrate the applicability/adaptability of PHYRN for phylogenetic inference of ancient protein families.

## Methods

### Collection and expansion of sequences

169 sequences used in Lin *et al.*[16] were collected and recA/RAD51 domain boundaries were defined using NCBI CDD default settings [21]. Homologous regions thus defined were used as query set for expansion. PSI-BLAST [22] was used to collect homologous (recA/RAD51 domain containing) sequences from NCBI NR database with an e-value threshold of 1e^-6^ with 3 iterations of profile-based search. The top 10% scoring hits of expansion results from each sequence were retained. After removing redundancy, the final data set was comprised of the 545 sequences. Furthermore, we used PHYRN to align 195 metagenomic sequences from Wu *et al.*[17] against the 545 recA-specific PSSM library. Based on the PHYRN composite score, these sequences were clustered using Pearson’s correlation and hierarchical clustering as available in Cluster 3.0 [23]. Next, 88 sequences belonging to ID2 (PSAR1), ID5 (PSAR2), ID4 (PUvsX), ID15 (Unknown 1), ID 11 (RecA-SAR1) and ID9 (Unknown 2) clusters were added into the previously described 545-sequence data set. For the sake of clarity and transparency, the sequence distribution of Set-1 and Set-2 reported above, as well as orthologous and paralogous pairwise comparisons reported in Table 1, do not include a set of 14 sequences. These were removed during dataset curation as they disrupted both the cladistic separation in subsampled trees and their unambiguous classification by phylogenetic analyses. These sequences are reported in Table 1 Legend. Although we have reason to believe that these sequences do belong to the recA/RAD51 superfamily [24], they need further analysis and validation.

**Table 1:** Qualitative and quantitative analysis of 17 sub-groups within the Reca/RAD51 superfamily

**Groups**	**No. of seq**	**Viruses**	**Meta-GOS**	**Bacteria**	**Archea**	**Eukarya**	**Pairwise % identity (ave in/btw groups)**
**recA**	**243**		**✓**	**✓**		**Pr, Fu, Pl,**	**61.5| 24.7**
**RADA**	**48**				**✓**		**56.8| 30.0**
**RADB**	**31**				**✓**		**44.0| 30.0**
**RADAB**	**5**				**✓**		**74.5| 30.0**
**DMC1**	**55**					**Pr, In, Nm, Fu, Pl, Ch**	**59.2| 29.9**
**RAD51**	**70**					**Pr, In, Nm, Fu, Pl, Ch**	**68.7| 29.6**
**RAD51C**	**24**					**Pr, Pl, Ch**	**51.5| 30.0**
**RAD51B**	**15**					**Pl, Ch (Pr)**	**51.4| 30.0**
**RAD51D**	**18**					**Pl, Ch (Pr, Fu, In)**	**48.7| 30.0**
**XRCC2**	**15**					**Pl, Ch (Pr, In)**	**46.6| 30.0**
**XRCC3**	**21**					**Pl, Ch (Pr, In)**	**48.9| 30.0**
**recA-SAR1**	**10**		**✓**				**74.6| 30.0**
**Phage SAR1**	**14**	**✓**	**✓**				**66.5| 30.0**
**Phage SAR2**	**17**	**✓**	**✓**				**73.3|30.0**
**Phage UvsX**	**21**	**✓**	**✓**				**66.6|30.0**
**Unknown 1**	**6**		**✓**				**67.4|30.0**
**Unknown 2**	**20**		**✓**		**✓**		**57.1|30.0**

Abbreviations are as follows: Protists (Pr), Insects (In), Nematodes (Nm), Fungi (Fu), Plants (Pl), and Chordate (Ch). Parentheses in RAD51B, D and XRCC2, XRCC3 groups denote species which are putative members of the respective group but were not included in the phylogenetic inference because they disrupt the overall topology and cannot be unambiguously assigned. These 14 sequences are listed below along with their GI numbers and species names.

XRCC2_303290256_Micromonas_pusilla_Plants, XRCC2_332024988_Acromyrmex_echinatior_Insecta, XRCC2_255074101_Micromonas_Plants, XRCC2_66803939_Dictyostelium_discoideum_Protists, XRCC2_281210087_Polysphondylium_pallidum_Protists, RAD51D_170071670_Culex_quinquefasciatus_Insecta, RAD51D_321474080_Daphnia_pulex_Animal, RAD51D_111226459_Dictyostelium_discoideum_Protist, XRCC3_307191609_Harpegnathos_saltator_Insecta, XRCC3_281201100_Polysphondylium_pallidum_Protist, XRCC3_170044836_Culex_quinquefasciatus_Insecta, XRCC3_307171500_Camponotus_floridanus_Insecta, RAD51B_45685353_Chlamydomonas_reinhardtii_Protists, ID9_Unknown2_118195642_Cenarchaeum_symbiosum_Protists.

### Implementation of PHYRN for recA/RAD51 sequences

The pipeline for the PHYRN algorithm is described in detail in Bhardwaj *et al.*[18]. The recA/RAD51 domain boundaries were defined in the full-length sequences using NCBI CDD with default settings [21]. These homologous regions were extracted using a custom python script and were used to generate a recA-specific PSSM library using codes provided in PHYRN v1.6 package (http://code.google.com/p/phyrn/). To increase the specificity of the PSSM library, we first collected all putative recA/RAD51 containing proteins, and subsequently used these sequences as a target database for pssmgen script in the PHYRNv1.6 package. Previous results with PHYRN have shown that an e-value of 1e^-6^ provides the best results with the non-redundant (NR) NCBI database [18]. Since our target recA/RAD51 database is significantly smaller in size, and the e-value threshold scales are proportional to the size of target database, we used an e-value of 7e^-13^ for PSSM generation. In the next step, full-length sequences were aligned with this PSSM library, and these alignments were encoded in a composite score matrix. While running rpsBLAST, we used a “–b” value setting that shows alignments for only the top scoring 75% of total PSSMs. In experiments with ROSE-derived synthetic protein families we validated that “–b” equal to 75% of total PSSMs provides the most accurate results. This composite score matrix was further used to calculate a Euclidean distance matrix. The Neighbor-Joining (NJ) algorithm as implemented in MEGA v5.03 [25] was used to calculate phylogenetic trees from the Euclidean distance matrix.

### Implementation of MSA/Protdist/ML

Optimal multiple sequence alignment (MSA) was calculated using MUSCLE v3.8 [26] with default settings. Protdist from PHYLIP package v3.69 [27,28] was used to calculate evolutionary distances. We used MEGA v5.03 to calculate the best protein substitution model for distance calculation. Based on these calculations, we used protdist with JTT (Jones, Taylor and Thornton) [29] as a substitution matrix of choice, and a gamma correction value of 0.8. For maximum likelihood (ML) trees, we used RAxML v7.2.8 [19] with MUSCLE alignment as input. RAxML was used with JTT as the substitution matrix of choice. Empirical frequencies were estimated from the data in hand (+E setting), and a gamma correction value 0.8 was used. All other settings were used as defaults.

### Statistical resampling

Statistical support for PHYRN was calculated using Jacknife resampling, while for protdist and ML trees Bootstrap resampling was used. For Jacknife resampling of PHYRN data, 80% of data points were randomly subsampled without replacement from the PHYRN NXM matrix. 5000 random replicates were generated in this manner and the Neighbor program from PHYLIP package [27,28] was used to calculate Neighbor-Joining trees. The Consense program from PHYLIP package [27,28] was used with the majority rule consensus method to calculate a consensus tree of 5000 replicates; these isometric consensus trees are shown in collapsed version and fully extended trees are available as supporting information (Additional file 2 Figure S2 & Additional file 3 FigureS3). The confidence values we obtained were compared for three-points of reference in the PHYRN trees, and were appended to branch labels in our PHYRN trees wherever appropriate (Figures 2&3). The symbol (-) denotes an unsupported branch in the tree. For protdist and ML method, Bootstrap resampling was conducted using their default settings with 1000 and 100 replicates respectively (Additional file 4 Figure S4 & Additional file 5 Figure S5).

**Figure 1:**
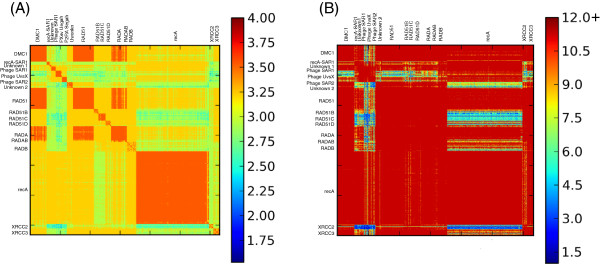
**Distribution and Characterization of PHYRN-Derived Phylogenetic Signal in recA/RAD51 Superfamily.** (**A**) Distribution of PHYRN Phylogenetic signal (%identity x %coverage) for recA/RAD51 superfamily. PHYRN score is calculated from alignments between full length query sequences and the respective recA/RAD51-specific PSSM library. PHYRN scores are represented as log-scaled values ranging from 0 (blue) to 4 (red). (**B**) Graphical representation of PHYRN phylogenetic signal of recA/RAD51 sequences (signal) as compared to their randomized versions (i.e. noise, 100 replicates). Comparative analysis is represented as Difference Ratio (DR).

**Figure 2:**
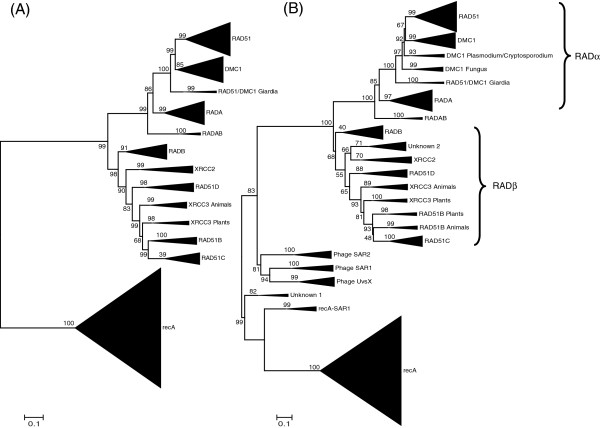
**Phylogenetic Inference of the recA/RAD51 Superfamily using PHYRN-NJ.** (**A**) Unrooted phylogram of recA/RAD51 clades of Set-1 of 545 sequences. (**B**) Unrooted phylogram of recA/RAD51 clades of Set-2 of 633 sequences (comprising of Set-1 + 88 metagenomic sequences). Confidence values are calculated by Jackknife resampling for 5000 replicates for both the sets. Scale bar is proportional to PHYRN-derived Euclidean distance scaled between 0-1.

### Randomization test for PHYRN-derived difference ratio

We conducted a randomization test to quantify a signal-to-noise ratio in our measurements of sequence homology. In this test, each full-length query sequence was randomized in its linear order of amino acids without replacement. Randomized sequences were then aligned with our recA-specific PSSM library and alignment scores were encoded in a new NXM-random data matrix. This randomization step was repeated for 100 different random replicates and an average and standard deviation for each coordinate was recorded. A Difference Ratio (DR) was calculated for each coordinate using the following equation and represented as log-scaled values:

(1) $$\mathrm{Difference}\;\mathrm{Ratio}=\frac{\left(\mathrm{composite}\;{\mathrm{score}}_{\mathrm{wt}}-\mathrm{average}\;\mathrm{composite}\;{\mathrm{score}}_{\mathrm{random}}\right)}{{\mathrm{SD}}_{\mathrm{random}}.}$$

Difference Ratio measures the tendency of full-length sequences to randomly align with domain specific PSSM library. Thus, Difference Ratio is a measure of specificity within the pairwise alignments, and quantifies the alignment score that could result due to random alignment for the particular query-PSSM pair.

## Results

### Construction of recA/RAD51 data sets

Our initial data set was comprised of 169 sequences that were obtained from Lin *et al.*[16]; this data set was expanded in number and diversity using PSI-BLAST [22] against the non-redundant NR NCBI database (see Methods). After this expansion, we obtained 545 sequences, denoted as Set-1. To obtain direct comparisons with the Wu *et al.*[17] study, we included 88 metagenomic sequences isolated from the Sorcerer II Global Ocean Sampling Expedition (GOS) [30], termed here Set-2. In Table 1, we present qualitative and quantitative statistics for both data sets, including the number and distribution of sequences in each sub-group of the recA/RAD51 family. For groups with sequences representative of eukaryotic lineages, we have further annotated the sequence diversity to demarcate the presence of protist, insect, nematode, fungi, plant, and/or chordate species. Phage SAR1, Phage SAR2 and Phage UvsX are enterobacteriophage sequences. We identified an archaea specific group, RADAB, which shows a split recombinase domain with the presence of a large insertion. With respect to sequence similarity, Set-1 and Set-2 are conserved within orthologous groups, but are divergent between paralogous groups (~30% average pairwise identity between groups as measured by MUSCLE [26], see Table 1). All sequences utilized in this study, as well as the chopped boundaries utilized for PSSM generation, are available upon request.

### Quantification of PHYRN difference ratio within the recA/RAD51 superfamily

Since all sequences in Set-1 and Set-2 share a common recA domain, these homologous domains were used to construct a recA/RAD51 specific PSSM library (see [18] and Methods for complete description of PHYRN implementation). Subsequently, full-length sequences from each data set were aligned with their respective recA/RAD51 PSSM library. The results from these alignments were collected and the alignment statistics (i.e. composite score = percentage identity X percentage coverage) were encoded as an N-query by M-PSSM (NXM) similarity matrix. The heat map in Figure 1A represents the phylogenetic signal of the NXM matrix for Set-2 represented on a log scale (red = maximal possible log score, 4; dark blue = lowest possible log score, 0). These data suggest that all sub-families have excellent signal within their group, and a varying amount of signal across paralogous sub-families.

**Figure 3:**
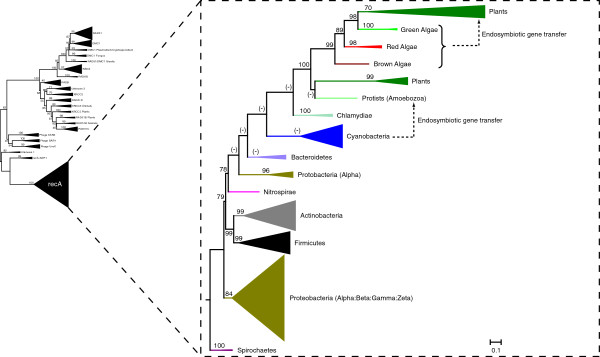
**Evolution of recA sequences.** (**A**) A phylogenetic tree of 242 recA sequences inferred using PHYRN-NJ and rooted with Spirochaetes. Branch statistics are derived from Jacknife resampling tests. The notation (-) is indicative of no support for the given branching pattern. Scale bar is proportional to PHYRN-derived Euclidean distance scaled between 0–1.

To further quantify the signal-to-noise ratio we conducted a randomization test, in which each full-length query sequence was randomized in its linear order of amino acids, without replacement, insuring that it retained the same length and amino acid composition. Randomized sequences were then aligned with the respective wild-type recA-specific PSSM library and alignment scores were encoded in a new NXM-random data matrix. This process was repeated for 100 different random replicates and an average and standard deviation for each coordinate was recorded. A Difference Ratio (DR) was calculated for each coordinate using Equation 3 (see Methods). Hence, the DR is a reflection of the amount of signal above background inherent to each comparison. The DR is plotted as a heat map in Figure 1B (blue = lowest SD above random, red = largest SD above random). We observed a strong signal-to-noise ratio across all the groups. Notably, metagenomic sequences also show strong signal against other groups, thereby justifying their inclusion in this phylogenetic study.

### Phylogenetic Inference of the recA/RAD51 Family

Unrooted phylogenetic trees for both Sets (Figures 2A &2B, respectively) were constructed from a Euclidian Distance of the NXM composite score matrix to produce an NXN distance matrix. Subsequently, a phylogenetic tree was inferred by distance-based NJ algorithm as described previously [31]. In the tree of Set-1, we observe three major clades, namely: (i) recA (ii) RADα and (iii) RADβ (see Figure 2A). Upon close inspection, the branching pattern is largely in accordance with Lin *et al*. [16]; however, there are some notable differences. Specifically: (i) we identified a new archaeal group, RADAB, between RADA and RADB archaea groups, (ii) we were able to include more representatives from protist, insect, nematode, archaea and bacterial sources across different clades, and (iii) our tree displays more robust statistical support across deep branches.

Between both sets, we also observed distinctive branching points at several positions. In the PHYRN-NJ tree of Set-1, ancestral RAD51/DMC1 Giardia sequences are outgroups to both DMC1 and RAD51 (DMC1 and RAD51 were monophyletic in Lin *et al.*). The presence of both DMC1 and RAD51 members in Plasmodium (chromoalveolate) suggests that duplication events leading to the origins of DMC1 from a common ancestor of DMC1 and RAD51 most likely happened after the evolution of alveolates (i.e. “with cavities”, a major line of protists). In the PHYRN-NJ tree of Set-2, fungal sequences seem to be misplaced, as there are ascomycetes (i.e. commonly called “sac fungi” or “cup fungi” for their cup-shaped fruiting bodies) both before and after the alveolates. Conversely, the PHYRN-NJ tree from Set-1 shows a clear demarcation of DMC1-fungal and RAD51-fungal sequences. It is possible that the addition of metagenomic sequences may have led to a decreased resolution of these specific groups. Another difference between PHYRN-based inferences of Set-2 is that XRCC2 occupies a phylogenetic position closer to the archaeal ancestors with high statistical support. Finally, XRCC3 forms a paraphyletic group (i.e. metazoans [animals] outgroup viridaeplantae [green plants] members). This could be due to a PHYRN-NJ branching error or a result of a differential evolutionary rate of XRCC3 between plants and animals.

Wu *et al.*[17] identified several new putative members of recA/RAD51 sequences from metagenomic sources. It is possible that the inclusion of these sequences would further refine our understanding of the deep origin of recA/RAD51 family. Indeed, inclusion of the metagenomic sequences (Figure 2B) leads to topological and statistical changes when compared to the tree inferred for Set-1 (compare Figure 2A to Figure 2B). Interestingly, the metagenomic groups occupy divergent positions in the tree. In fact, Unknown 1 attains the most basal position in our PHYRN-NJ tree. In both our present study and that of Lin *et al*. [16], RADα and RADβ share a common ancestor. This is in contrast to the study of Wu *et al*. [17] and is a more parsimonious scenario assuming a recA/Unknown 1 root.

We also observe that endosymbiotic transfer events from bacterial recAs contributed to the evolution of eukaryotic recA proteins (Figure 3). Specifically, multiple gene transfer events from cyanobacteria and chlamydiae (i.e. obligate intracellular pathogens AKA ‘energy parasites’) led to the evolution of chloroplast recAs. This is in accordance with the literature on the origins of chloroplast [32-35]. We also observe another clade of viridaeplantae members that shows closer relationships with protist members. These recA sequences are nuclear in location, and may represent nuclear localized copies of endosymbiotic DNA, or may be products of secondary or tertiary endosymbiosis involving protist members. Moreover, our study infers that Gram positive bacteria (Actinobacteria and Firmicutes) form sister taxa in rooted trees.

Finally, we compared the PHYRN-NJ tree shown in Figure 2B to phylogenies inferred using multiple sequence alignment-based methods (Additional file 4 Figure S4 & Additional file 5 Figure S5). Notably, both Muscle-NJ and Muscle-RAxML trees show similar positioning of metagenomic groups as compared to PHYRN-NJ; however, the Muscle-NJ tree shows lesser statistical support when compared to Muscle-RAxML and PHYRN-NJ trees. Importantly, the Muscle-RAxML tree predicts a non-parsimonious branching pattern for RADα and RADβ. Specifically, in the Muscle-RAxML tree, RADβ clades show a closer relationship with recA, whereas RADα clades evolve from RADβ clades (Additional file 5 Figure S5). Domain analysis, functional relationships and previous studies show that this scenario is highly unlikely [36-40]. Studies on functional characterization of RADα have shown, that their roles in homologous recombination are similar to the function of bacterial recA, while RADβ shows significant functional divergence and innovation from bacterial recA [36,41]. Thus, it is more plausible that gene duplication events in recA gave rise to RADα and RADβ in eukaryotes and archaea, such that RADα retained similar functions, while the RADβ group evolved to gain new functions. Furthermore, in the RAxML tree RAD51 Giardia sequences appear after the emergence of more complex mammalian DMC1 & RAD51 members, which presents an unlikely scenario. Hence, we believe that the evolutionary scenario presented by the MUSCLE-RAxML tree is not a likely occurrence, and is not well supported by the functional studies of RADα and RADβ.

A PHYRN-NJ analysis provides a more refined, statistically robust, and logical phylogenetic inference for this data. However, even the PHYRN-NJ tree lacks resolution at some nodes, specifically for the events occurring after the emergence of Unknown 2 (archaea) and before the diversification of RAD51 groups (XRCC2, XRCC3, RAD51B-D). Hence, the inclusion of metagenomic sequences leads to a loss of resolution and robustness with respect to the DMC1 and RAD51B lineages. Also, in the PHYRN-NJ tree, there are some possible topological errors, such as the position of fungal DMC1 sequences, even though it receives strong statistical support in the resampling analysis. These types of errors might be a function of: (i) missing sequences in the metagenomic groups, (ii) missing protists, nematodes, fungi, or insect sequences in higher-order groups that we could not find or could not include in the tree (see Table 1), (iii) possible sequencing errors for some representatives, (iv) branching errors by NJ, and/or (v) inaccurate distance estimates by PHYRN for some sequences.

## Discussion

**Figure 4:**
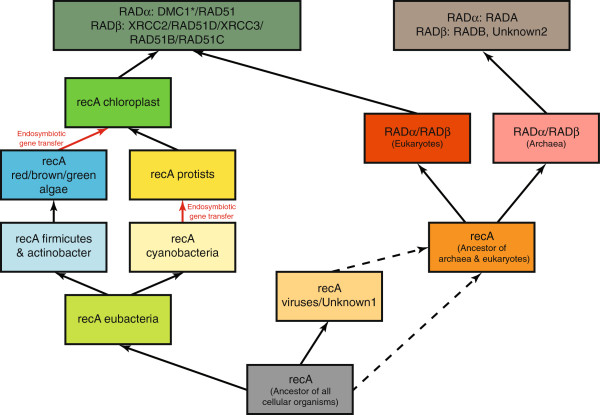
**Model of the Evolutionary History of the recA/RAD51 Superfamily.** Graphical representation of a model for evolution of recA/RAD51 family based on the phylogenetic trees obtained using PHYRN methodology. Endosymbiotic gene transfer events from cyanobacteria to protists and algae to plants are labeled. (*) represents Meiosis specific gene.

We present a PHYRN-based phylogenetic inference for recA/RAD51, an ancient family of DNA repair proteins. Our results suggest that this phylogeny is more refined/resolved than previous reports considering our: (i) more comprehensive data set including older and metagenomic sequences, (ii) more parsimonious evolutionary scenario, and (iii) significant signal over noise ratio and larger statistical support across the entire landscape of protein representatives, despite the high levels of sequence divergence. Based on the PHYRN-derived phylogenetic trees, we propose a scenario for the evolution of recA/RAD51 family of proteins (Figure 4). In this model, we make inferences on a number of key points, including: (i) the ancient origins of recA, (ii) differential rates of evolution for recA/RAD51 subfamilies, and (iii) the role(s) of endosymbiotic gene transfer events in the evolution of eukaryotic recA.

In our current model, the earliest recA evolved in a common ancestor of eubacteria and Unknown1 group. Regarding recA, we infer multiple gene transfer events from cyanobacteria leading to the evolution of chloroplast recA, in accordance with the origin of chloroplasts from cyanobacterial ancestors [32]. Based on the position and mutational rates of Unknown 1, our study corroborates the divergent nature of Unknown 1. Moreover, recA-SAR1 likely represents an intermediate group between Unknown1 and known eubacterial clades (i.e. recA). Interestingly, the inferred rates of evolution in recA-SAR1 are very different from all other eubacterial clades, and are similar to evolutionary rates exhibited by members of Unknown1.

It is well accepted that subsequent gene duplication events led to the diversification of ancient recA to RADα and RADβ in archaea and eukaryotes [16,17]. Our study also identifies an intermediate archaeal group (RADAB) between RADA and RADB. Interestingly, both RADB and RADAB show monophyletic groups with members from the class euryarcheota, whereas RADA shows members from both major classes of archaea (i.e. crenoarcheota and euryarcheota). Within the RADA lineage, further gene duplications in protists presumably led to diversification of function into: (i) meiosis-specific DMC1 and (ii) RAD51, which have both somatic DNA repair and meiosis-specific genes. As a result of this taxonomic diversity, it is likely that DMC1 evolved in old alveolate members. Moreover, it is possible that DMC1 in higher eukaryotes attained a more specialized meiosis-specific role through multiple loss of functional mutations over time. In the RADB lineage, we propose, in contrast to Wu *et al*. [17], that Unknown 2 attains a position closer to RADB. Given that both these groups are archaea-specific this positioning is more plausible. Furthermore, we infer at least two gene duplications in archaea: eukaryotic RAD51D, XRCC3, RAD51B and RAD51C evolved as a result of the first duplication while eukaryotic XRCC2 might have evolved in a second gene duplication event in RADB lineage.

Overall, through the use of large taxon sampling and PHYRN methodology, we have provided a robust phylogenetic inference of recA/RAD51 superfamily. Our previous studies with synthetic data sets have shown that PHYRN provides accurate phylogenetic inference even in highly divergent data sets. However, PHYRN is an MSA-independent distance based method, and like all distance-based methods, it might be prone to extreme among-site rate variation. We still need to explore the effect of long-branch attraction issues on PHYRN performance. In many cases, increased taxon sampling may overcome issues arising due to long-branch attraction, and we have collected a comprehensive data set of recA/RAD51 proteins in this study. In future studies, we will explore methods to further refine PHYRN, and will include measures that quantify the effect of rate heterogeneity and long-branch attraction on PHYRN performance and accuracy.

## Conclusions

Comprehensively, this study makes a number of contributive advances: (i) we present further validation of PHYRN-based inference in an ancient protein family with variable rates, and (ii) we derive a refined model of recA/RAD51 evolution. Finally, we corroborate the notion put forth by Wu *et al.*[17] and concur that annotation of more metagenomic recA sequences and their inclusion in the phylogenetic inference is essential for a deeper and more refined understanding of recA/RAD51 phylogeny and endosymbiotic transfer events in general.

## Competing interests

The authors declare that they have no competing interests.

## Authors’ contributions

SVC, GB, DBV and RLP planned the project. SVC, GB, DBV & RLP developed and implemented the methods along with the interpretation, analyzing the dataset and writing the manuscript. JB, YH and ZZ helped in generating the intermediate programming codes for PHYRN software. LH, CAB, GKT, XZ, HM, AA participated in collection and performing the experiments. All authors read and approved the final manuscript.

## Supplementary Material

Additional file 1: Figure S1 Phylogenetic Inference of the recA/RAD51 Superfamily using MSA-based methods. Representative phylogenetic trees of recA/RAD51 gene family as inferred in (A) Lin *et al.* (2006) and (B) Wu *et al.* (2011). Clades with metagenomic sequences that are unique to Wu *et al.* are demarcated in red. The notation (-) is indicative of no support for the given branching pattern. Click here for file

Additional file 2: Figure S2 Uncollapsed PHYRN tree of 545-recA/RAD51 sequences (Set-1). Phylogram of 545 recA/RAD51 sequences as inferred using PHYRN. Euclidean distance was calculated using a 545 x 545 composite score matrix, and trees were calculated from Euclidean distance matrix using Neighbor-Joining (NJ) algorithm. Confidence values were calculated using Jacknife resampling of 5000 replicates, wherein 80% of the matrix was subsampled for each replicate. Click here for file

Additional file 3: Figure S3 Uncollapsed PHYRN tree of 633-recA/RAD51 sequences (Set-2). Phylogram of 633 recA/RAD51 sequences as inferred using PHYRN. Euclidean distance was calculated using a 633 x 633 composite score matrix, and trees were calculated from Euclidean distance matrix using Neighbor-Joining (NJ) algorithm. Confidence values were calculated using Jacknife resampling of 5000 replicates, wherein 80% of the matrix was subsampled for each replicate. [The metagenomic sequences added in 6 new groups have retained the same ID numbers presented in Wu et. al. (ID15- Unknown 1, ID2- Phage SAR1, ID5-Phage SAR2, ID4-Phage UvsX, ID11-recA-SAR1 and ID9-Unknown 2)]. Click here for file

Additional file 4: Figure S4 Phylogenetic Inference of recA/RAD51 protein family inferred using MUSCLE-NJ. Phylogenetic tree of 633 recA/RAD51 sequences as inferred using MUSCLE-NJ. Optimal MSA was obtained using MUSCLE. Protdist from PHYLIP v 3.9 was used to calculate distance matrix with JTT as substitution matrix of choice, and gamma value of 0.8. Confidence values were calculated using Bootstrap resampling method with 1000 replicates. Click here for file

Additional file 5: Figure S5 Collapsed MUSCLE-RaxML tree of 633-recA/RAD51 sequences.Phylogenetic tree of 633 recA/RAD51 sequences as inferred using MUSCLE-RaxML. Optimal MSA was obtained using MUSCLE. Protdist from PHYLIP v 3.9 was used to calculate distance matrix with JTT as substitution matrix of choice, and gamma value of 0.8. Confidence values were calculated using Bootstrap resampling method with 1000 replicates. Click here for file
