# Supplementary figures and images for: Reevaluation of the evolutionary events within recA/RAD51 phylogeny
Source: BMC Genomics. 2013 Apr 10;14:240. doi: 10.1186/1471-2164-14-240 (PMC3637515; doi:10.1186/1471-2164-14-240)

(A)

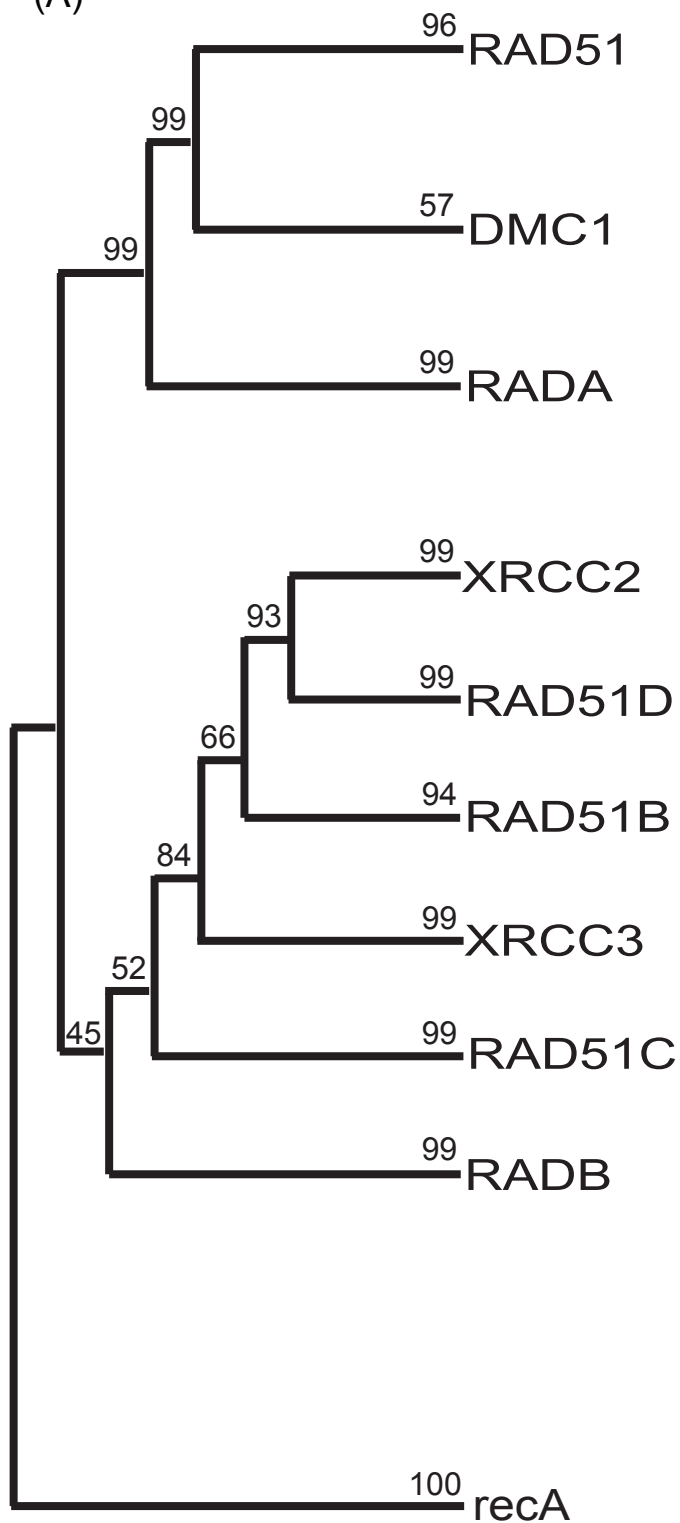

(B)

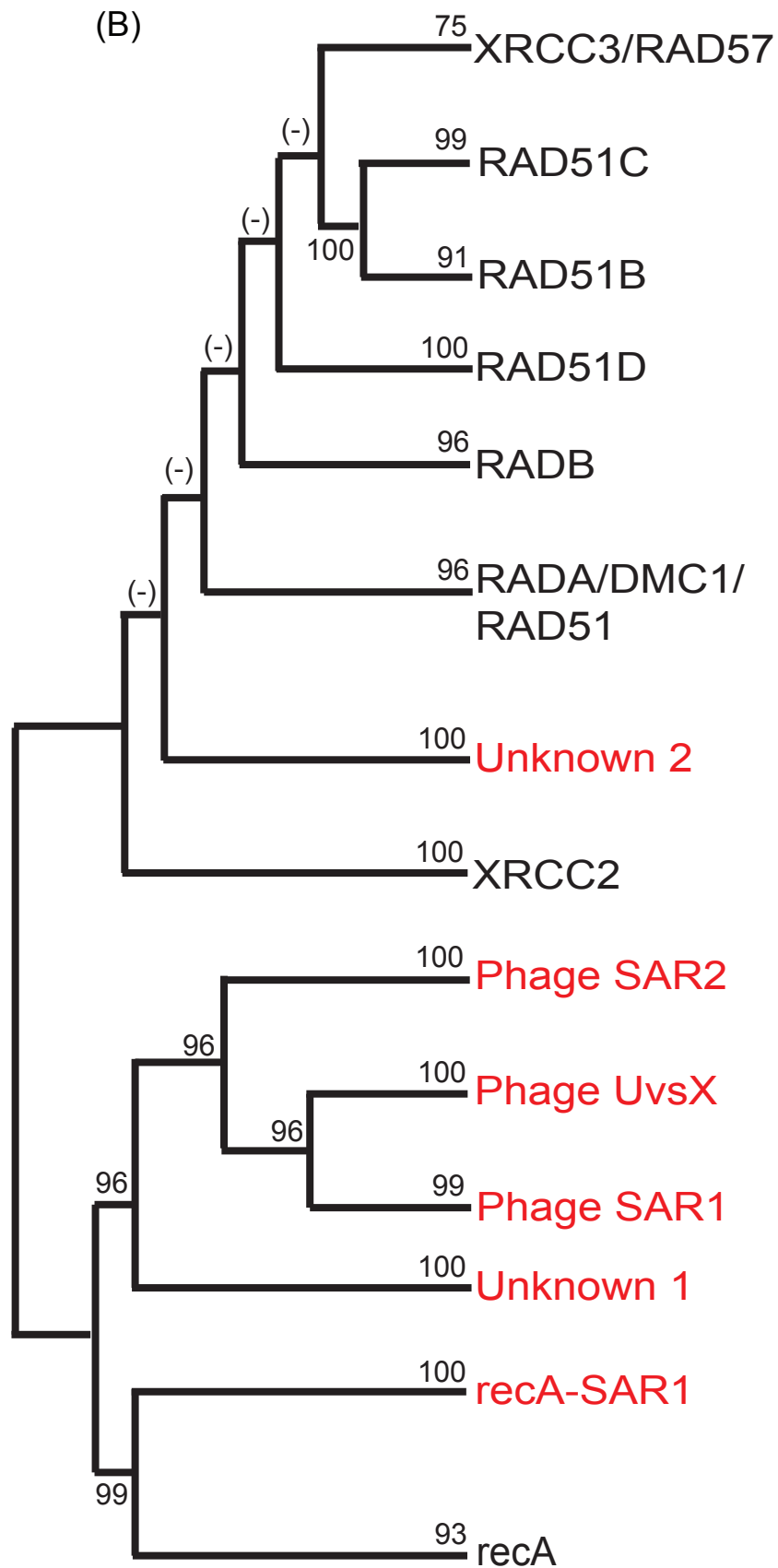

Supplement: Additional file 1: Figure S1 — Phylogenetic Inference of the recA/RAD51 Superfamily using MSA-based methods. Representative phylogenetic trees of recA/RAD51 gene family as inferred in (A) Lin et al. (2006) and (B) Wu et al. (2011). Clades with metagenomic sequences that are unique to Wu et al. are demarcated in red. The notation (-) is indicative of no support for the given branching pattern. [file 1471-2164-14-240-S1.pdf]

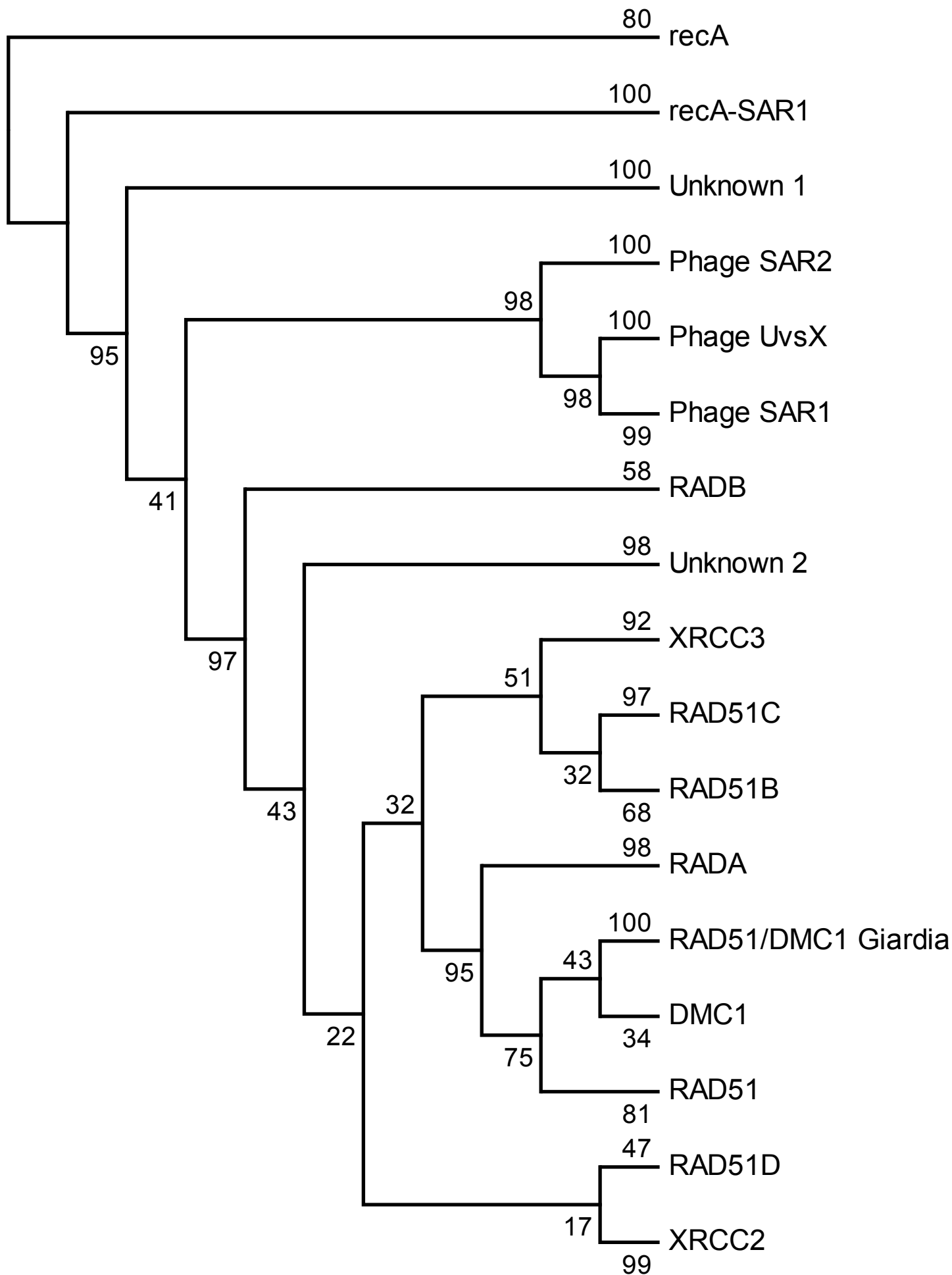

Supplement: Additional file 4: Figure S4 — Phylogenetic Inference of recA/RAD51 protein family inferred using MUSCLE-NJ. Phylogenetic tree of 633 recA/RAD51 sequences as inferred using MUSCLE-NJ. Optimal MSA was obtained using MUSCLE. Protdist from PHYLIP v 3.9 was used to calculate distance matrix with JTT as substitution matrix of choice, and gamma value of 0.8. Confidence values were calculated using Bootstrap resampling method with 1000 replicates. [file 1471-2164-14-240-S4.pdf]

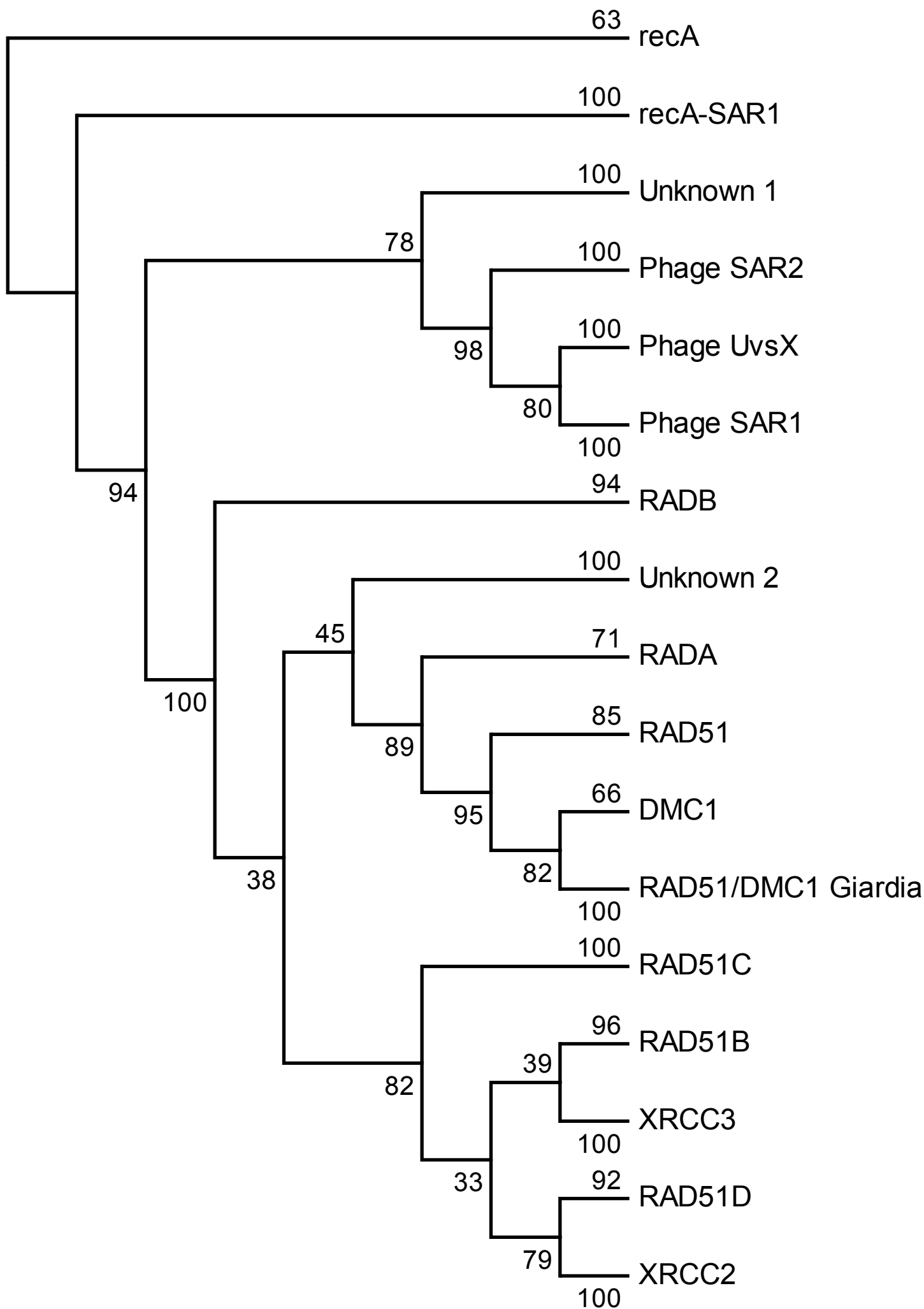

Supplement: Additional file 5: Figure S5 — Collapsed MUSCLE-RaxML tree of 633-recA/RAD51 sequences.Phylogenetic tree of 633 recA/RAD51 sequences as inferred using MUSCLE-RaxML. Optimal MSA was obtained using MUSCLE. Protdist from PHYLIP v 3.9 was used to calculate distance matrix with JTT as substitution matrix of choice, and gamma value of 0.8. Confidence values were calculated using Bootstrap resampling method with 1000 replicates. [file 1471-2164-14-240-S5.pdf]
