# Supplementary material for: Reevaluation of the evolutionary events within recA/RAD51 phylogeny
Source: BMC Genomics. 2013 Apr 10;14:240. doi: 10.1186/1471-2164-14-240 (PMC3637515; doi:10.1186/1471-2164-14-240)

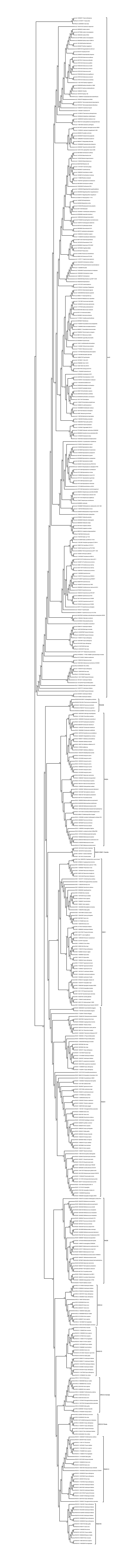

Supplement: Additional file 2: Figure S2 — Uncollapsed PHYRN tree of 545-recA/RAD51 sequences (Set-1). Phylogram of 545 recA/RAD51 sequences as inferred using PHYRN. Euclidean distance was calculated using a 545 x 545 composite score matrix, and trees were calculated from Euclidean distance matrix using Neighbor-Joining (NJ) algorithm. Confidence values were calculated using Jacknife resampling of 5000 replicates, wherein 80% of the matrix was subsampled for each replicate. [file 1471-2164-14-240-S2.pdf]
