# Supplementary material for: Reevaluation of the evolutionary events within recA/RAD51 phylogeny
Source: BMC Genomics. 2013 Apr 10;14:240. doi: 10.1186/1471-2164-14-240 (PMC3637515; doi:10.1186/1471-2164-14-240)

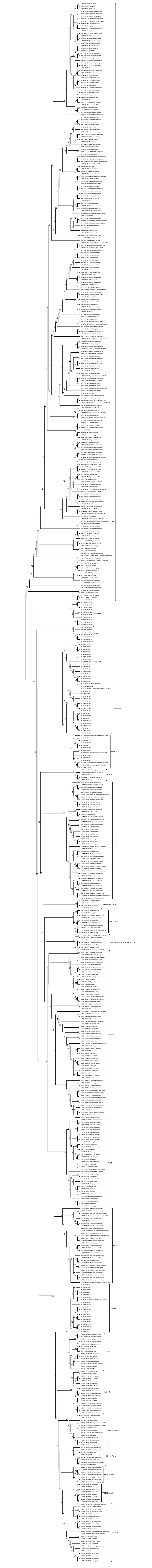

Supplement: Additional file 3: Figure S3 — Uncollapsed PHYRN tree of 633-recA/RAD51 sequences (Set-2). Phylogram of 633 recA/RAD51 sequences as inferred using PHYRN. Euclidean distance was calculated using a 633 x 633 composite score matrix, and trees were calculated from Euclidean distance matrix using Neighbor-Joining (NJ) algorithm. Confidence values were calculated using Jacknife resampling of 5000 replicates, wherein 80% of the matrix was subsampled for each replicate. [The metagenomic sequences added in 6 new groups have retained the same ID numbers presented in Wu et. al. (ID15- Unknown 1, ID2- Phage SAR1, ID5-Phage SAR2, ID4-Phage UvsX, ID11-recA-SAR1 and ID9-Unknown 2)]. [file 1471-2164-14-240-S3.pdf]
